# Environmental Dependence of Artifact CD Peaks of Chiral Schiff Base 3d-4f Complexes in Soft Mater PMMA Matrix

**DOI:** 10.3390/ijms12106966

**Published:** 2011-10-19

**Authors:** Yu Okamoto, Keisuke Nidaira, Takashiro Akitsu

**Affiliations:** Department of Chemistry, Faculty of Science, Tokyo University of Science, 1–3 Kagurazaka, Shinjuku-ku, Tokyo 162-8601, Japan

**Keywords:** chirality, solid state CD, soft mater, schiff base complexes

## Abstract

Four chiral Schiff base binuclear 3d-4f complexes (NdNi, NdCu, GdNi, and GdCu) have been prepared and characterized by means of electronic and CD spectra, IR spectra, magnetic measurements, and X-ray crystallography (NdNi). A so-called artifact peak of solid state CD spectra, which was characteristic of oriented molecules without free molecular rotation, appeared at about 470 nm. Magnetic data of the complexes in the solid state (powder) and in PMMA cast films or solutions indicated that only GdCu preserved molecular structures in various matrixes of soft maters. For the first time, we have used the changes of intensity of artifact CD peaks to detect properties of environmental (media solid state (KBr pellets), PMMA cast films, concentration dependence of PMMA in acetone solutions, and pure acetone solution) for chiral 3d-4f complexes (GdCu). Rigid matrix keeping anisotropic orientation exhibited a decrease in the intensity of the artifact CD peak toward negative values. The present results suggest that solid state artifact CD peaks can be affected by environmental viscosity of a soft mater matrix.

## 1. Introduction

Organic/inorganic hybrid materials containing photochromic organic dyes and transition metal complexes such as models of chiral catalysts [1–3] and magnetic materials [4–8] have been developed widely aiming at photo-induced switching systems as supramolecules. As hybrid materials are essentially mixtures, overlapped strong absorption bands of π-π * and n-π * regions make it difficult to deal with individual components generally. To overcome this problem, we employed polarized electronic spectra [9–12] and polarized IR spectra [13,14] in order for selective detection of molecular orientation, or fluorescence spectra [15] as well as absorption spectra. Only CD spectra of chiral components can be observed selectively for hybrid materials of chiral and achiral ones, which show overlapped absorption spectra. Additionally, so-called artifact peaks may sometimes be a serious problem for solid state CD spectra [16–31], in which chiral molecules take anisotropic orientation without a degree of freedom for molecular motion. Although this problem has mainly been dealt with in view of improvement to reduce noise of CD spectra in the solid state, investigation in soft mater matrixes (intermediate between isotropic solutions and anisotropic solids) or positive applications for obtaining useful information have not been carried out so far.

By the way, chiral 3d-4f Schiff base complexes affording interesting structures [32–34] may also be appropriate candidates for distinguishable transitions for separate observation of CD bands. The analogous 3d-4f complexes have been investigated being focused on magnetic properties [35,36] or emission [37–39] resulting from 4f-electrons of lanthanide ions and their interactions to 3d electrons of transiton metal ions. It is well known that detailed treatment of superexchange interactions of 3d-4f molecule-based magnets is difficult because of spin-orbit interactions except for Gd(III) complexes. In order to overcome this, empirical estimation of magnetic interactions between 3d-4f ions are sometimes carried out. Although this may also be interesting for chiroptical or other CD spectroscopic studies, few studies have been reported to date.

**Figure 1:**
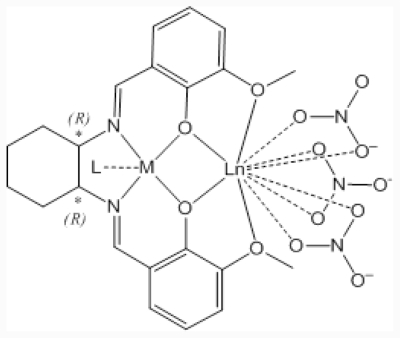
Molecular structures of complexes (NdNi, NdCu, GdNi, and GdCu denote Ln and M metal ions). Crystalline water molecules were omitted for clarity.

In this paper, we report on preparations and characterization of new chiral Schiff base binuclear 3d-4f complexes, NdNi, NdCu, GdNi, and GdCu (Figure 1), including optical properties of 3d-4f moieties. We also prepared PMMA cast films containing these complexes and confirmed only the structure of GdCu was kept by means of magnetic measurement. We measured CD spectra of GdCu in a pure acetone, as acetone solutions of PMMA (different concentrations), and in the solid state (KBr pellets). Comparison with solid state and complete solution suggested so-called artifact peaks of solid state CD spectrum. As far as we know, we attempt to use the intensity of artifact CD peaks exhibiting environmental dependence of soft mater matrixes for the first time.

## 2. Results and Discussion

### 2.1. Crystal Structure of NdNi

**Figure 2:**
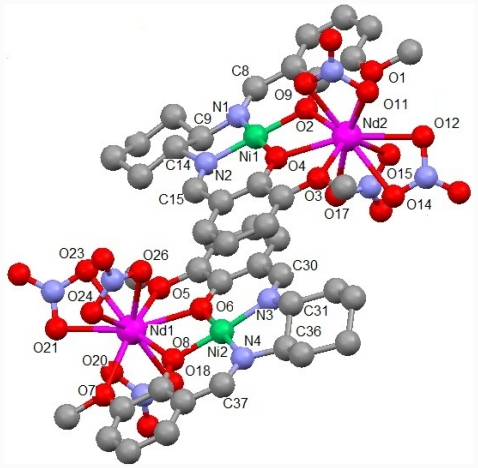
Crystal structures of NdNi showing selected atom labeling scheme. Hydrogen atoms are omitted clarity.

Typical reactions of Schiff base ligands, 3d (Ni(II) or Cu(II), and Zn(II)) ions, and 4f (Nd(III) or Gd(III)) ions gave rise to analogous binuclear 3d-4f complexes [39]. Since Nd(III) ion can coordinate to this ligand more rigidly than Gd(III) ion generally, which results in different features of crystal growth and dissociation in solutions. As depicted in Figure 2, the crystal structure could be determined only for NdNi (the other complexes must be similar binuclear structures). Unfortunately, suitable single crystals could not be obtained for other complexes. Asymmetric unit of NdNi contains two crystallographically independent molecules which are coupled with π-π stacking of ligands by noncovalent interactions. Therefore, intramolecular superexchange interactions between 3d and 4f ions can be expected in view of crystal structures (see later section). Ni(II) and Nd(III) ions afford a square planar *trans*-[NiN_2_O_2_] and ten-coordinated prism-like [GdO_10_] coordination environment, as summarized in Table 1. Ni(II) ion coordinates no additional axial ligands. Each bond distance and angle of the ligand moieties including cyclohexyl groups connected to asymmetric carbon atoms of (*R*,*R*)-configuration are within normal values for analogous Schiff base complexes [32,33,39].

### 2.2. Magnetic Properties

**Figure 3:**
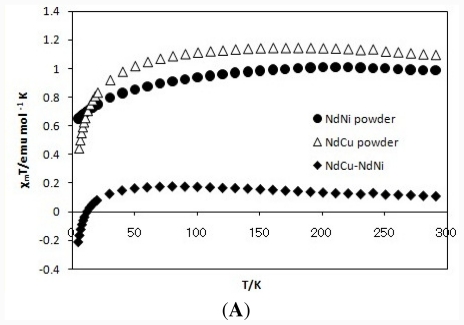

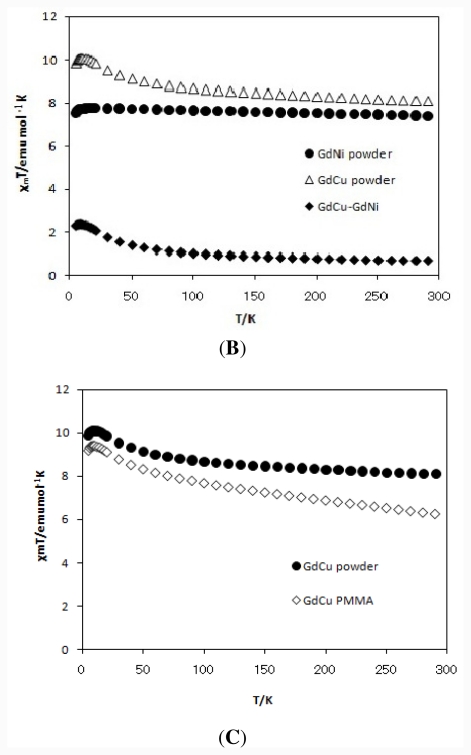
(**A**) The *χ_M_T* vs *T* plots for NdNi and NdCu as powder and the differences of *χ*_M_T(NdCu-NdNi) at 0.5 T; (**B**) The *χ_M_T* vs *T* plots for GdNi and GdCu as powder and the differences of *χ*_M_T(GdCu-GdNi) at 0.5 T; (**C**) The *χ_M_T* vs *T* plots for GdCu as powder and as a PMMA cast film at 0.5 T.

Figures 3A and Figure 3B show the *χ_M_T* vs *T* plots of Nd(III) (NdNi and NdCu) and Gd(III) (GdNi and GdCu) complexes including the differences of *χ_M_T* values (*χ_M_T*(NdCu)-*χ_M_T*(NdNi) and *χ_M_T*(GdCu)-*χ_M_T*(GdNi)). As the data at 300 K suggested, Cu(II) ion and Ni(II) ion of four-coordinated square planar coordination environment are S = 1/2 (paramagnetic) and S = 0 (diamagnetic), respectively. The ground electronic state of Nd(III) ion is ^4^I_9/2_ with S = 3/2, L = 6, J = 9/2 with λ = 290 cm^−1^, while that of Gd(III) ion is ^8^S_7/2_ with S = 7/2, L = 0, J = 7/2 with λ = 0 cm^−1^, the total orbital angular momentum is zero and single ion anisotropy due to spin-orbit interaction should be negligible. Therefore, empirical proof of ferromagnetic superexchange interaction based on the differences of *χ_M_T* values may be clear and reliable. Actually, the corresponding data of Nd(III) complexes (*χ_M_T*(NdCu)-*χ_M_T*(NdNi)) did not exhibit increasing of low-temperature region, while that of Gd(III) complexes (*χ_M_T*(GdCu)-*χ_M_T*(GdNi)) exhibited typical peak in the low-temperature region. Therefore, the 3d-4f binuclear moiety of GdCu can be judged to be ferromagnetic one.

Figure 3C shows comparison of the *χ_M_T vs T* plots of GdCu as a bulk powder sample (identical to the data above) and GdCu as PMMA cast films. Contrary to other three complexes (not shown), only GdCu exhibited similar behavior regardless of bulk and film except for increasing of diamagnetic contribution of PMMA in the high-temperature region. The results suggest that coordination of lanthanide ion is kept only for GdCu. In this way, GdCu is suitable for the flowing discussion associated with CD spectroscopy because of these two conditions, namely, ferromagnetic interaction and rigidity of Gd(III) ion coordination.

### 2.3. CD Spectra in the Solid State

**Figure 4:**
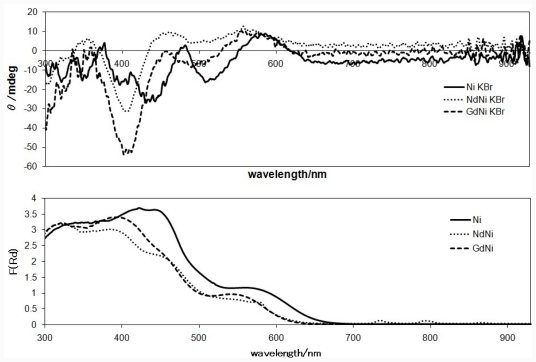
The CD and diffuse reflectance electronic spectra of NdNi and GdNi (and the corresponding mononuclear Ni complex for comparison) measured as KBr pellets.

**Figure 5:**
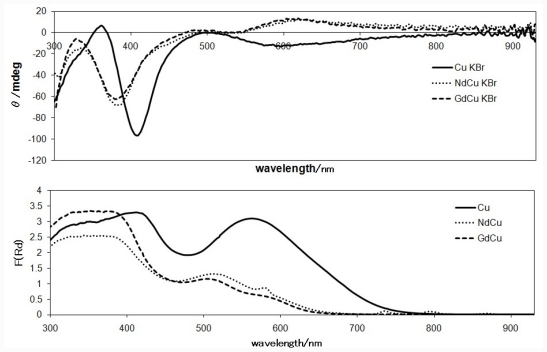
The CD and diffuse reflectance electronic spectra of NdCu and GdCu (and the corresponding mononuclear Cu complex for comparison) measured as KBr pellets.

Figures 4 and 5 show solid state CD spectra and the corresponding diffuse reflectance electronic spectra of Ni(II) complexes (NdNi, GdNi, and the related mononuclear Ni complex having the identical chiral ligand) and Cu(II) complexes (NdCu, GdCu, and mononuclear Cu), respectively. The different features of d-d band around 500–600 nm for mononuclear Ni and Cu complexes are ascribed to conventional difference of square planar Schiff base Ni(II) and Cu(II) complexes. Indeed, the signs of the corresponding CD bands are opposite. When Nd(III) ions are introduced, sharp f-f peaks appeared at 580, 740, and 790 nm in the diffuse reflectance electronic spectra. When Nd(III) or Gd(III) ions are introduced or replaced, the region of charge transfer bands are affected predominantly. The corresponding CD bands are changed; in particular, the region of 500–550 nm for Ni(II) complexes exhibited clear shifts, which is not clear in the diffuse reflectance electronic spectra. When comparing this with the CD spectra in solutions (in which free molecular motion is permitted), so-called artifact CD peaks in the solid state (in which molecular motion is not permitted completely) can be assigned to the peaks around 470 nm, which is neighboring region of d-d bands around 500–600 nm and an important region for chiroptical features of binuclear 3d-4f systems.

### 2.4. CD Spectra in PMMA Films or Solutions

**Figure 6:**
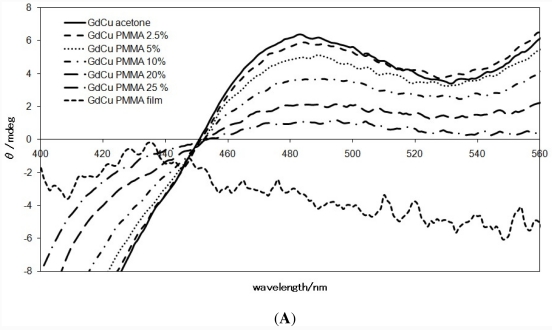

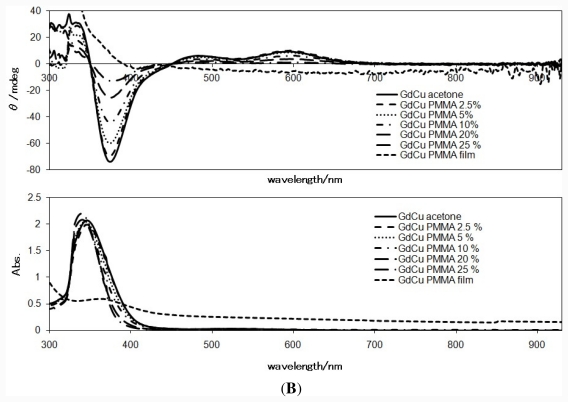
(**A**) Concentration (viscosity) dependence of the CD spectra of PMMA film, PMMA acetone solutions, and acetone solution of GdCu (magnified); (**B**) Concentration (viscosity) dependence of CD and the corresponding electronic absorption spectra of PMMA film, PMMA acetone solutions, and acetone solution of GdCu.

Figure 6B shows the CD and the corresponding electronic absorption spectra of GdCu measured as PMMA cast films, PMMA acetone solutions of various concentration (mass%), and acetone solution. PMMA cast film is a novel environment, having flexibility in which molecular orientation can be kept and simultaneously molecular motion (conformational changes) can be permitted. Furthermore, acetone solutions with a high concentration of PMMA lose its freedom of molecular motion. In these solutions, anisotropy of molecular orientation gradually may be increased to appear so-called artifact CD peaks, which is ascribed to losing isotropy of molecular orientation in rigid environment of a matrix. Little changes of the corresponding electronic absorption spectra also support that spectral changes of CD spectra are essentially ascribed to not reactions or changes of molecules but the principle of CD spectroscopy.

Figure 6A is magnification of the CD spectra (Figure 6B). It indicates concentration or viscosity-dependence of the CD spectra of PMMA acetone solutions of GdCu. As PMMA cast film and solutions, intensity changes (negative decreasing of *θ* values) with a CD peak around 470 nm assigned as an artifact CD peak of solid state indicated, depending on viscosity of matrix. In this way, the intensity of so-called artifact peaks is changed as the degree of restriction of molecular motion in correlation with concentration of viscous PMMA concentration or environment of a soft mater or complete solid state.

## 3. Experimental Section

### 3.1. General Procedures

Chemicals of the highest commercial grade available (solvents from Kanto Chemical, organic compounds from Tokyo Chemical Industry and metal sources from Wako) were used as received without further purification.

### 3.2. Preparations

#### 3.2.1. Preparation of NdNi

To a solution of *o*-vanillin (0.3069 g, 2.00 mmol) dissolved in methanol (40 mL), (*1R*,*2R*)-(−)-1,2-cyclohexandiamine (0.1231 g, 1.00 mmol) was added dropwise and stirred at 313 K for 2 h to give yellow solution of ligand. Nickel(II) acetate tetrahydrate (0.2493 g, 1.00 mmol) was added to give rise to orange solution of the complex. After stirring for 2 h, neodymium (III) nitrate hexahydrate (0.4552 g, 1.00 mmol) was added and refluxed at 373 K for 4 h to yield orange solution with orange precipitates. This crude orange compound was filtered and recrystallized from methanol/diethyl ether to give orange prismatic single crystals suitable for X-ray analysis containing solvents. Yield 0.5804 g (89.9%). IR (KBr (cm^−1^)): 451, 565, 667, 740, 748, 785, 856, 965, 1031, 1078, 1166, 1232, 1307, 1381, 1468, 1624(C=N), 1652, 1699, 2413, 2848, 2927. UV-vis (nm (Abs)): (acetone) 332 (0.96). (methanol) 331 (0.46). (chloroform) 339 (0.73). CD (nm (*θ*/mdeg)): (acetone) 394 (−25.5), 450 (4.66), 568 (5.47). (methanol) 391 (−10.2), 456 (1.22), 559 (3.13). (chloroform) 404 (−5.42), 459 (1.03), 566 (1.51).

#### 3.2.2. Preparation of NdCu

To a solution of *o*-vanillin (0.3054 g, 2.00 mmol) dissolved in methanol (40 mL), (*1R*,*2R*)-(−)-1,2-cyclohexandiamine (0.1171 g, 1.00 mmol) was added dropwise and stirred at 313 K for 2 h to give yellow solution of ligand. Copper (II) acetate monohydrate (0.2048 g, 1.00 mmol) was added to give rise to purple-red solution of the complex. After stirring for 2 h, neodymium (III) nitrate hexahydrate (0.4493 g, 1.00 mmol) was added and refluxed at 373 K for 4 h to yield red solution with red precipitates. This crude red compound was filtered and recrystallized from methanol/diethyl ether to give red precipitates. Yield 0.6333 g (97.4%). IR (KBr (cm^−1^)): 400, 433, 560, 642, 740, 759, 849, 950, 1022, 1072, 1223, 1280, 1380, 1458, 1603, 1628(C=N), 1652, 1699, 2412, 2843, 2942. UV-vis (nm (Abs)): (acetone) 346 (2.37). (methanol) 347 (0.30). (chloroform) 358 (0.52). CD (nm (*θ*/mdeg)): (acetone) 374 (−91.5), 490 (7.61), 598 (11.9). (methanol) 372 (−11.4), 491 (0.92), 606 (2.27). (chloroform) 389 (−25.4), 483 (2.21), 605 (4.32).

#### 3.2.3. Preparation of GdNi

To a solution of *o*-vanillin (0.3124 g, 2.00 mmol) dissolved in methanol (40 mL), (*1R*,*2R*)-(−)-1,2-cyclohexandiamine (0.1358 g, 1.00 mmol) was added dropwise and stirred at 313 K for 2 h to give yellow solution of ligand. Nickel (II) acetate tetrahydrate (0.2517 g, 1.00 mmol) was added to give rise to orange solution of the complex. After stirring for 2 h, gadolinium (III) nitrate hexahydrate (0.4640 g, 1.00 mmol) was added and refluxed at 373 K for 4h to yield orange solution with orange precipitates. This crude orange compound was filtered and recrystallized from methanol/diethyl ether to give orange precipitates. Yield 0.5859 g (89.0%). IR (KBr (cm^−1^)): 419, 452, 565, 667, 739, 784, 856, 1033, 1076, 1164, 1232, 1308, 1380, 1471, 1508, 1619(C=N), 1652, 1699, 2412, 2831, 2928. UV-vis (nm (Abs)): (acetone) 331 (1.86). (methanol) 331 (0.46). (chloroform) 340 (0.65). CD (nm (*θ*/mdeg)): (acetone) 392 (−44.5), 444 (8.65), 555 (10.1). (methanol) 397 (−6.34), 458 (1.08), 555 (1.91). (chloroform) 404 (−13.6), 459 (2.41), 574 (3.55).

#### 3.2.4. Preparation of GdCu

To a solution of *o*-vanillin (0.3022 g, 2.00 mmol) dissolved in methanol (40 mL), (*1R*,*2R*)-(-)-1,2-cyclohexandiamine (0.1143 g, 1.00 mmol) was added dropwise and stirred at 313 K for 2 h to give yellow solution of ligand. Copper (II) acetate monohydrate (0.2089 g, 1.00 mmol) was added to give rise to purple-red solution of the complex. After stirring for 2 h, gadolinium (III) nitrate hexahydrate (0.4634 g, 1.00 mmol) was added and refluxed at 373 K for 4 h to yield red solution with red precipitates. This crude red compound was filtered and recrystallized from methanol/diethyl ether to give red precipitates. Yield 0.5220 g (78.0%). IR (KBr (cm^−1^)): 430, 560, 667, 740, 758, 838, 949, 1020, 1072, 1165, 1222, 1303, 1374, 1460, 1527, 1605, 1624, 1652, 2420, 2842, 2924. UV-vis (nm (Abs)): (acetone) 346 (2.06). (methanol) 346 (2.04). (chloroform) 360 (0.79). CD (nm (*θ*/mdeg)): (acetone) 375 (−73.9), 483 (6.39), 569 (9.46). (methanol) 374 (−62.8), 487 (5.28), 604 (12.4). (chloroform) 397 (−35.6), 481 (3.47), 609 (1.94).

### 3.3. Physical Measurements

Infrared spectra were recorded as KBr pellets on a JASCO FT-IR 4200 plus spectrophotometer in the range of 4000–400 cm^−1^ at 298 K. Absorption (and diffuse reflectance) electronic spectra were measured on a JASCO V-570 UV/VIS/NIR spectrophotometer (equipped with an integrating sphere) in the range of 800–200 nm at 298 K. Circular dichroism (CD) spectra were measured on a JASCO J-820 spectropolarimeter in the range of 800–200 nm at 298 K. Magnetic properties were investigated with a Quantum Design MPMS-XL superconducting quantum interference device magnetometer (SQUID). DC magnetic susceptibility data were measured at 5–300 K under 5000 Oe (measured at Institute for molecular Science and Institute for Solid State Physics, the University of Tokyo).

### 3.4. X-ray Crystallography

Orange prismatic single crystals of NdNi was glued on top of a glass fiber and coated with a thin layer of epoxy resin to measure the diffraction data. Intensity data were collected on a Bruker APEX2 CCD diffractometer with graphite monochromated Mo K*α* radiation (*λ* = 0.71073 Å). Data analysis was carried out with a SAINT program package. The structures were solved by direct methods with a SHELXS-97 [40] and expanded by Fourier techniques and refined by full-matrix least-squares methods based on *F^2^* using the program SHELXL-97 [40]. An empirical absorption correction was applied by a program SADABS. All non-hydrogen atoms were readily located and refined by anisotropic thermal parameters. All hydrogen atoms were located at geometrically calculated positions and refined using riding models. Unfortunately, appropriate single crystals could not be obtained for other metal complexes.

Crystallographic data for NdNi. C_22_H_24_N_5_NdNiO_13_, crystal size 0.15 mm × 0.13 mm × 0.12 mm, M_w_ = 769.41, triclinic, space group *P*1, *a* = 9.3035(11) Å, *b* = 12.1272(14) Å, *c* = 12.2122(14) Å, *α* = 101.6500(10) °, *β* = 96.2000(10) °, *γ* = 90.0350(10) °, *V* = 1341.2(3) Å ^3^, *Z* = 2, *D_calc_* = 1.905 mg/m ^3^, F(000) = 766, R_1_ = 0.0224, wR_2_ = 0.0675 (6137 reflections), *S* = 0.582, Flack parameter = −0.005(13). (where R_1_ = ∑||F_o_|−|F_c_||/∑|F_o_|. R_w_ = (∑w(|F_o_|−|F_c_|)^2^ /∑w|F_o_|^2^)^1/2^, w = 1/(σ ^2^(F_o_) + (0.1P)^2^), P = (F_o2_ + 2F_c2_)/3).

## 4. Conclusions

In summary, among four chiral 3d-4f complexes reported herein, only GdCu was confirmed to be suitable for CD spectroscopy experiments in various environmental matrixes. In comparison with acetone solution and solid state CD spectra, the bands at about 470 nm in the solid state spectra could be assigned as a so-called artifact CD peak in the solid state. In this PMMA polymer matrixes system, that suggests anisotropic molecular orientation as well as restricted molecular motion, we have shown matrix viscosity-dependence of the intensity of artifact peaks resulting from chiroptical features of 3d-4f complexes for the first time. Rigid matrix maintaining anisotropic orientation exhibited a decrease in intensity of the artifact CD peak toward negative *θ* values.

## Supplementary Data

CCDC 837663 contains the supplementary crystallographic data. These data can be obtained free of charge via http://www.ccdc.cam.ac.uk/conts/retrieving.html, or from the Cambridge Crystallographic Data Centre, 12 Union Road, Cambridge CB2 1EZ, UK; fax: +44-1223-336-033; or E-Mail: deposit@ccdc.cam.ac.uk.

## Figures and Tables

**Table 1:** Selected bond distance (Å) and angles (°) of NdNi.

Nd1-O6 = 2.374(7)	Nd1-O8 = 2.377(6)	Nd1-O20 = 2.468(8)	Nd1-O18 = 2.470(7)
Nd1-O23 = 2.477(7)	Nd1-O21 = 2.482(7)	Nd1-O26 = 2.510(7)	Nd1-O24 = 2.514(7)
Nd1-O5 = 2.528(6)	Nd1-O7 = 2.543(6)	Nd1-N8 = 2.873(8)	Nd1-N9 = 2.902(8)
Nd2-O2 = 2.337(7)	Nd2-O4 = 2.395(7)	Nd2- O11 = 2.463(9)	Nd2-O9 = 2.478(7)
Nd2-O15 = 2.504(7)	Nd2-O1 = 2.507(6)	Nd2-O14 = 2.518(6)	Nd2-O3 = 2.522(7)
Nd2-O12 = 2.523(7)	Nd2-O17 = 2.533(9)	Nd2-N5 = 2.921(11)	Nd2-N7 = 2.942(9)
Ni1-O4 = 1.838(6)	Ni1-N2 = 1.843(7)	Ni1-N1 = 1.844(8)	Ni1-O2 = 1.876(7)
Ni2-O8 = 1.833(6)	Ni2-N4 = 1.849(8)	Ni2-N3 = 1.855(8)	Ni2-O6 = 1.861(7)
O4-Ni1-N2 = 94.3(3)	O4-Ni1-N1 = 175.9(3)	N2-Ni1-N1 = 88.7(3)	O4-Ni1-O2 = 81.9(3)
N2-Ni1-O2 = 175.8(3)	N1-Ni1-O2 = 95.2(3)	O8-Ni2-N4 = 95.3(3)	O8-Ni2-N3 = 175.7(3)
N4-Ni2-N3 = 85.9(3)	O8-Ni2-O6 = 82.8(3)	N4-Ni2-O6 = 175.3(3)	N3-Ni2-O6 = 96.3(3)
O6-Nd1-O8 = 61.9(2)	O6-Nd1-O20 = 117.0(3)	O8-Nd1-O20 = 118.6(2)	O6-Nd1-O18 = 73.6(3)
O8-Nd1-O18 = 74.9(2)	O20-Nd1-O18 = 51.4(3)	O6-Nd1-O23 = 110.7(2)	O8-Nd1-O23 = 144.0(2)
O20-Nd1-O23 = 96.5(2)	O18-Nd1-O23 = 139.6(2)	O6-Nd1-O21 = 161.2(2)	O8-Nd1-O21 = 134.3(2)
O20-Nd1-O21 = 66.8(3)	O18-Nd1-O21 = 116.6(3)	O23-Nd1-O21 = 51.0(2)	O6-Nd1-O26 = 68.1(2)
O8-Nd1-O26 = 77.1(2)	O20-Nd1-O26 = 164.3(2)	O18-Nd1-O26 = 139.9(2)	O23-Nd1-O26 = 68.1(2)
O21-Nd1-O26 = 103.5(2)	O6-Nd1-O24 = 111.9(2)	O8-Nd1-O24 = 77.6(2)	O20-Nd1-O24 = 130.3(2)
O18-Nd1-O24 = 144.6(2)	O23-Nd1-O24 = 73.3(2)	O21-Nd1-O24 = 69.8(3)	O26-Nd1-O24 = 50.2(2)
O6-Nd1-O5 = 63.2(2)	O8-Nd1-O5 = 124.0(2)	O20-Nd1-O5 = 78.2(2)	O18-Nd1-O5 = 80.4(2)
O23-Nd1-O5 = 68.0(2)	O21-Nd1-O5 = 101.7(2)	O26-Nd1-O5 = 92.4(2)	O24-Nd1-O5 = 134.3(2)
O6-Nd1-O7 = 123.8(2)	O8-Nd1-O7 = 64.4(2)	O20-Nd1-O7 = 76.1(2)	O18-Nd1-O7 = 77.9(2)
O23-Nd1-O7 = 122.2(2)	O21-Nd1-O7 = 74.8(2)	O26-Nd1-O7 = 114.3(2)	O24-Nd1-O7 = 70.2(2)
O5-Nd1-O7 = 153.2(2)	O6-Nd1-N8 = 97.3(2)	O8-Nd1-N8 = 95.4(2)	O20-Nd1-N8 = 25.7(2)
O18-Nd1-N8 = 26.1(3)	O23-Nd1-N8 = 120.5(2)	O21-Nd1-N8 = 90.8(2)	O26-Nd1-N8 = 165.4(2)
O24-Nd1-N8 = 141.1(2)	O5-Nd1-N8 = 81.3(2)	O7-Nd1-N8 = 72.3(2)	O6-Nd1-N9 = 137.1(2)
O8-Nd1-N9 = 145.1(2)	O20-Nd1-N9 = 81.6(2)	O18-Nd1-N9 = 132.8(3)	O23-Nd1-N9 = 26.4(2)
O21-Nd1-N9 = 24.6(2)	O26-Nd1-N9 = 85.2(2)	O24-Nd1-N9 = 68.0(2)	O5-Nd1-N9 = 86.1(2)
O7-Nd1-N9 = 97.3(2)	N8-Nd1-N9 = 107.3(2)	O2-Nd2-O4 = 61.9(2)	O2-Nd2-O11 = 119.4(3)
O4-Nd2-O11 = 118.3(3)	O2-Nd2-O9 = 76.9(2)	O4-Nd2-O9 = 72.9(3)	O11-Nd2-O9 = 52.0(3)
O2-Nd2-O15 = 79.1(2)	O4-Nd2-O15 = 114.1(2)	O11-Nd2-O15 = 127.0(3)	O9-Nd2-O15 = 147.3(2)
O2-Nd2-O1 = 62.8(2)	O4-Nd2-O1 = 121.8(2)	O11-Nd2-O1 = 75.8(3)	O9-Nd2-O1 = 78.6(2)
O15-Nd2-O1 = 70.7(2)	O2-Nd2-O14 = 141.3(3)	O4-Nd2-O14 = 111.1(2)	O11-Nd2-O14 = 97.8(3)
O9-Nd2-O14 = 140.3(2)	O15-Nd2-O14 = 69.5(2)	O1-Nd2-O14 = 122.9(2)	O2-Nd2-O3 = 123.2(2)
O4-Nd2-O3 = 61.9(2)	O11-Nd2-O3 = 81.3(3)	O9-Nd2-O3 = 79.7(3)	O15-Nd2-O3 = 132.5(2)
O1-Nd2-O3 = 155.2(2)	O14-Nd2-O3 = 69.5(2)	O2-Nd2-O12 = 134.9(2)	O4-Nd2-O12 = 160.91(19)
O11-Nd2-O12 = 65.1(3)	O9-Nd2-O12 = 116.2(3)	O15-Nd2-O12 = 68.2(3)	O1-Nd2-O12 = 77.2(2)
O14-Nd2-O12 = 50.7(2)	O3-Nd2-O12 = 101.9(2)	O2-Nd2-O17 = 74.6(3)	O4-Nd2-O17 = 67.3(3)
O11-Nd2-O17 = 166.0(3)	O9-Nd2-O17 = 138.5(3)	O15-Nd2-O17 = 51.6(2)	O1-Nd2-O17 = 113.1(3)
O14-Nd2-O17 = 68.3(3)	O3-Nd2-O17 = 91.3(3)	O12-Nd2-O17 = 105.3(2)	O2-Nd2-N5 = 96.9(3)
O4-Nd2-N5 = 95.2(3)	O11-Nd2-N5 = 27.2(3)	O9-Nd2-N5 = 24.8(3)	O15-Nd2-N5 = 143.0(3)
O1-Nd2-N5 = 74.8(3)	O14-Nd2-N5 = 121.8(3)	O3-Nd2-N5 = 80.5(3)	O12-Nd2-N5 = 91.6(3)
O17-Nd2-N5 = 162.5(3)	O2-Nd2-N7 = 76.9(2)	O4-Nd2-N7 = 92.1(3)	O11-Nd2-N7 = 149.4(3)
O9-Nd2-N7 = 153.6(2)	O15-Nd2-N7 = 25.0(3)	O1-Nd2-N7 = 92.0(3)	O14-Nd2-N7 = 65.1(2)
O3-Nd2-N7 = 112.7(2)	O12-Nd2-N7 = 85.0(2)	O17-Nd2-N7 = 26.7(3)	
